# Cerebral malaria—using the retina to study the brain

**DOI:** 10.1038/s41433-023-02432-z

**Published:** 2023-02-14

**Authors:** Nicholas A. V. Beare

**Affiliations:** Department of Eye and Vision Science, University of Liverpool, Liverpool, L7 8TX UK

**Keywords:** Parasitic infection, Infection, Outcomes research, Diseases of the nervous system

## Abstract

Cerebral malaria (CM) remains a common cause of death of children in Africa with annual mortality of 400 000. Malarial retinopathy is a unique set of fundus signs which has diagnostic and prognostic value in CM. Assessment of malarial retinopathy is now widely utilised in clinical care, and routinely incorporated into clinical studies to refine entry criteria. As a visible part of the central nervous system, the retina provides insights into the pathophysiology of this infectious small-vessel vasculitis with adherent parasitised red blood cells. Fluorescein angiography and optical coherence tomography (OCT) have shown that patchy capillary non-perfusion is common and causes ischaemic changes in the retina in CM. It is likely this is mirrored in the brain and may cause global neurological impairments evident on developmental follow up. Three types of blood-retina barrier breakdown are evident: large focal, punctate, and vessel leak. Punctate and large focal leak (haemorrhage in formation) are associated with severe brain swelling and fatal outcome. Vessel leak and capillary non-perfusion are associated with moderate brain swelling and neurological sequelae. These findings imply that death and neurological sequelae have separate mechanisms and are not a continuum of severity. Each haemorrhage causes a temporary uncontrolled outflow of fluid into the tissue. The rapid accumulation of haemorrhages, as evidenced by multiple focal leaks, is a proposed mechanism of severe brain swelling, and death. Current studies aim to use optic nerve head OCT to identify patients with severe brain swelling, and macula OCT to identify those at risk of neurological sequelae.

## Abstract (zh)

脑型疟疾 (CM) 仍为非洲儿童死亡的一个常见原因, 每年的死亡人数可达40万人。疟疾性视网膜病变为具有独特眼底体征的一系列改变, 对CM具有诊断和预后价值。在临床工作中, 对于疟疾性视网膜病变的评估已得到广泛应用, 并常规纳入临床研究以完善入选标准。作为中枢神经系统中唯一肉眼可见的组织, 视网膜提供了对这种寄生于红细胞的传染性小血管炎的病理生理学了解的有用信息。

荧光素血管造影和相干光断层扫描 (OCT) 显示, 斑片状毛细血管无灌注很常见, 并导致CM患者的视网膜缺血性改变。这些病理性改变可能反映在大脑中的病变, 并导致在发育的随访中出现了明显的的全神经系统损伤。三种类型的血-视网膜屏障破坏是显而易见的: 大面积病灶、点状病灶和血管渗漏。点状和大面积病灶渗漏 (出血形成) 与严重的脑组织肿胀和致命的临床解决有关。血管渗漏和毛细血管无灌注与中度脑组织肿胀和神经系统后遗症有关。这些发现意味着死亡和神经系统后遗症具有不同的机制, 并不是严重后遗症的结局。

每一次出血都会导致液体暂时不受控制地流出到组织中。多处病灶渗漏表明, 出血的快速积累, 是严重脑肿胀和死亡的可能机制。

目前的研究旨在使用视盘OCT来识别严重脑水肿的患者, 以及使用黄斑OCT来识别那些具有神经系统后遗症风险的患者。

## Introduction

Understanding cerebral malaria (CM) is manifestly important; malaria still kills 400 000 people a year and almost all of those are children in Africa with cerebral malaria [1]. But efforts at understanding the effect of CM on the central nervous system (CNS) microvasculature and its tissue, have been hampered by lack of access to the CNS in vivo. The ocular fundus, and retinal imaging in particular, offers a unique insight into the effect of CM on the CNS, how it causes coma, death and disability.

CM is caused by *Plasmodium falciparum*. After an infected mosquito bite, parasites go through a liver stage as sporozoites before merozoites are released into the blood to infect red blood cells (RBC), and asexually multiply within them as trophozoites consuming haemoglobin. Infected red cells are misshapen and express adhesion molecules enabling them to roll along and adhere to the vessel endothelium and each other. This adhesion of parasitised red blood cells (pRBC) within the vasculature is termed sequestration and is thought to be a mechanism whereby they evade clearance in the spleen. Trophozoites mature into schizonts which rupture the pRBC, releasing more merozoites. Sequestration is the histopathological hallmark of CM occurring in the brain and other organs [2]. How sequestration of pRBCs within the CNS microvasculature and associated effects lead to coma, convulsions, raised intracranial pressure (ICP), death and neurological injury is poorly understood [3]. Severe anaemia, metabolic acidosis and disseminated intravascular coagulation are also characteristics of CM.

Mortality of children who reach hospital with CM remains high, around 15–35% [4, 5], and most of these are within a few hours of admission. This is even after the introduction of intravenous artesunate which rapidly kills malaria parasites [5]. It is now clear from MRI brain studies of children with CM that severe brain swelling and inferred elevated ICP is critical to fatal outcome [6]. Coning of the brain stem through the foramen magnum is consistent with the mode of death which is usually respiratory arrest.

Disability in survivors was previously thought to be limited to the neurological deficits which were evident to clinical examination in 23% of surviving children at discharge [7]. However more detailed follow up studies have shown that as well as these gross neurological deficits up to 50% of survivors demonstrate developmental delay, epilepsy, and even behavioural problems a year after their CM episode [8, 9]. Identifying these children early, that is during the acute illness, would enable early intervention to the benefit of children and their parents [10].

The mortality rate of other causes of coma in children in Africa including, bacterial meningitis, viral encephalitis, rabies and sub-arachnoid haemorrhage, may be even higher than CM. However in areas where malaria is common (endemic), and brain imaging is absent, patients with these conditions may be falsely diagnosed with CM if they also have a malaria infection at the same time. In an autopsy study in Malawi 23% of patients diagnosed with CM had another cause of death identified and did not have significant sequestration [11].

These are the challenges for malaria researchers and clinicians who are treating patients for whom bednets, initial treatment or new vaccines have failed. These children present to health facilities with no brain imaging or sophisticated laboratory or microbiological testing. The parasite can be killed rapidly, but we need better ways to identify patients at risk of death and disability; and adjunctive therapies which can reduce these outcomes, and are implementable in poorly resourced settings.

## Malarial retinopathy

Malarial retinopathy is made up of specific and non-specific features. The specific features are retinal whitening (macular and peripheral whitening) and vessel abnormalities or discolouration. The non-specific features are blot haemorrhages which are mainly white-centred, papilloedema and cotton wool spots (Table 1) [12, 13].

**Table 1:** Fundus signs of Malarial Retinopathy, their imaging findings and pathogenesis.

Fundus sign	Sub-category	Imaging findings	Pathogenesis
Retinal whitening	Macular whitening	Capillary non-perfusion on FA. Inner retinal hyper-reflectivity on OCT	Retinal ischaemia secondary to sequestration of parasitised RBCs
	Peripheral whitening		
Vessel abnormalities	Orange vessels; white edges (tramlining) & lesions in blood column	Intravascular filling defects on FA. Hyper-reflective vessel walls and lumen seen on OCT	Sequestration of intact parasitised RBCs
	White vessels	Occluded vessels on FA	Fragmented RBCs, haemazoin & monocytes filling vessel lumens
	Capillary whitening	Occluded capillaries (and post-capillary venules) on FA. Hyper-reflective dots on OCT	
Haemorrhages	White-centred (predominantly)	Large focal leak on FA during formation, masking thereafter Well-circumscribed sub-ILM lesions, or deeper diffuse inner retina lesions on OCT	Uncertain. Fibrin and fibrinogen clot
	Blot		Uncertain
Papilloedema		Disk leak on FA Thickening of papillary RNFL on OCT	Raised intracranial pressure impeding axoplasmic flow
Cotton Wool Spots		Focal swelling of RNFL on OCT	Marker of retinal ischaemia

*FA* fluorescein angiography, *OCT* optical coherence tomography, *RBC* red blood cells, *ILM* inner limiting membrane, *RNFL* retinal nerve fibre layer.

### Macular whitening

**Fig. 1:**
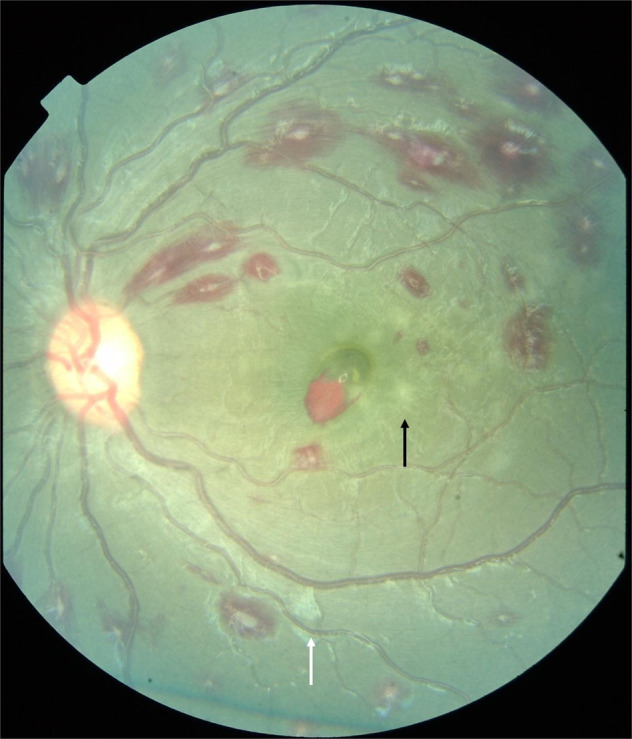
Malarial retinopathy. Features illustrated are white-centred haemorrhages, a superficial blot haemorrhage at the fovea, mild macular whitening (black arrow) and cotton wool spot (white arrow).

**Fig. 2:**
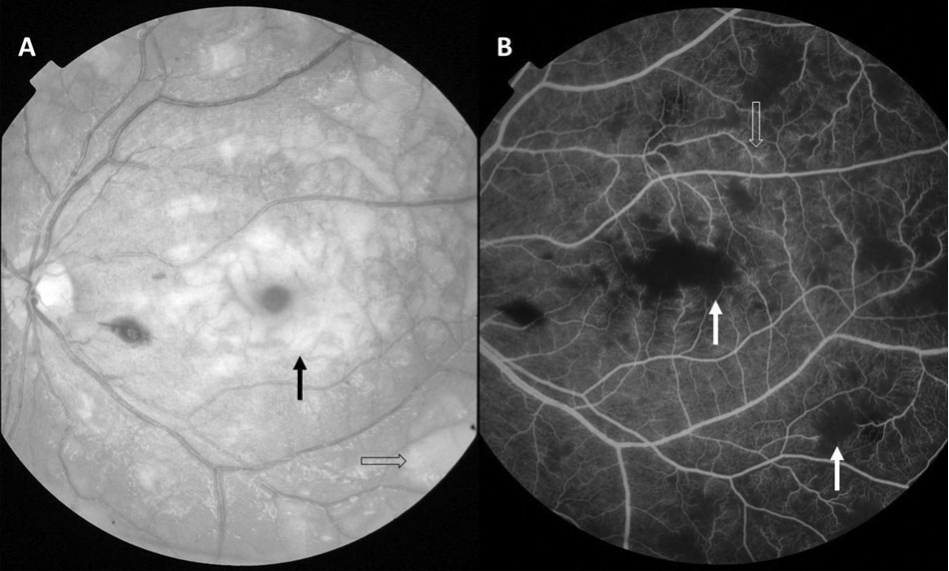
Malarial retinopathy. **A** Red-free image showing severe and extensive retinal whitening including macular whitening (black arrow) and peripheral whitening (open arrow). **B** Mid-phase fluorescein angiogram of the same patient showing corresponding capillary non-perfusion (white arrow), and vessel leak from post-capillary venules (open arrow).

**Fig. 3:**
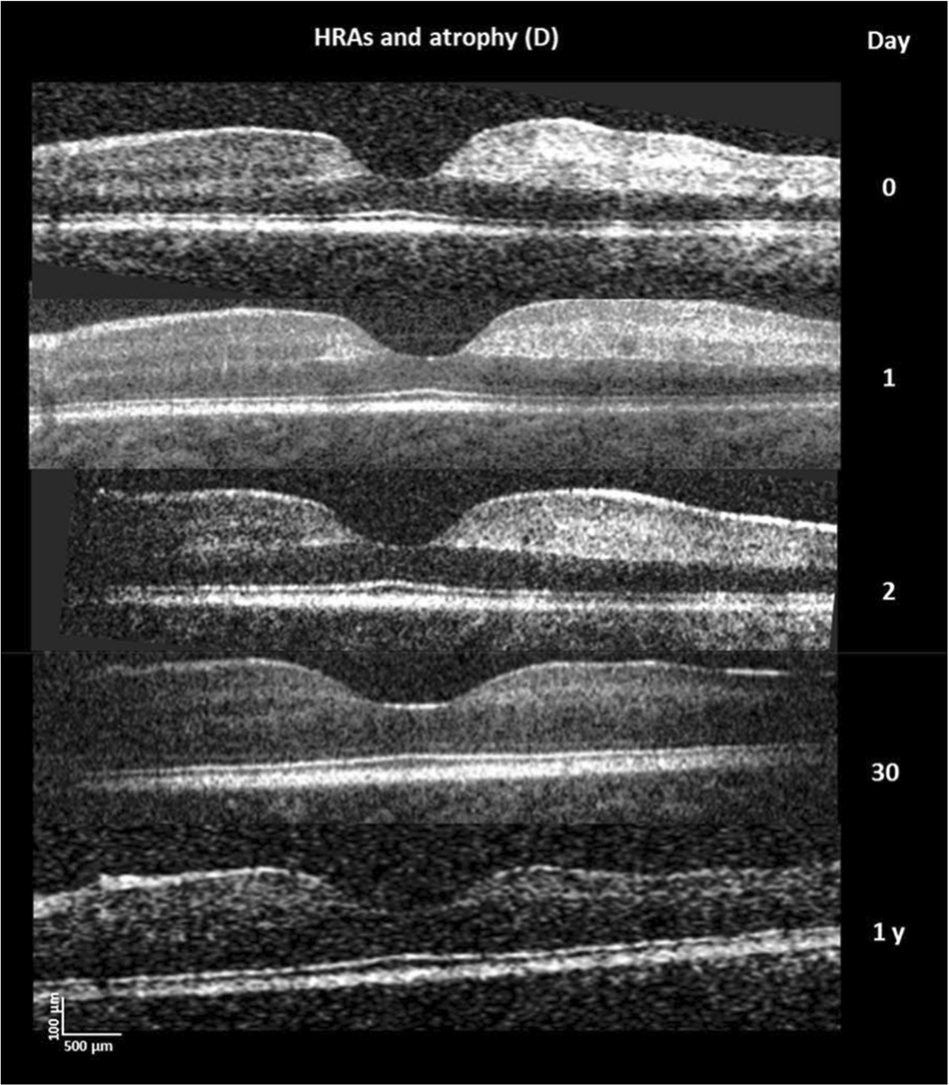
Macular OCT scans of 22-month-old patient with cerebral malaria. Successive OCT scans of macular whitening showing inner retinal hyper-reflective lesions on either side of the foveal dip and temporal macula (righthand side). At one-year retinal thinning is evident in the previous hyper-reflective area. Reproduced from Tu et al. Sci Rep. 2021 Aug 3;11:(1)15722. 10.1038/s41598-021-94495-9.

Macular whitening (MW) is a patchy whitening or opacification of the retina in the macula. It is seen predominantly around the fovea (sparing the foveola) and temporal raphe in areas of size less than 1/3 of a disc area to several disc areas in severe cases (Figs. 1 and 2). From studies including fluorescein angiography (FA) we know this to be an ischaemic change as it is directly contiguous with areas of capillary non-perfusion (Fig. 2) [14]. Investigations with handheld optical coherence tomography (OCT) demonstrate loss of structure and hyper-reflectivity of the inner retinal layers (Fig. 3) [15]. The outer retina is spared which is accounted for by the virtual absence of sequestration in the choroid.

### Peripheral whitening

Peripheral whitening (PW) can be a similar patchy whitening indicating an ischaemic opacification of the retina (Fig. 2). However, it can be a subtle more generalised mosaic whitening which magnification of fundus images has shown to be whitening of capillaries in some cases. Cotton wool spots occur but are whiter and more superficial than retinal whitening (Fig. 1). OCT has shown that cotton wool spots in the nerve fibre layer are more common than has been recognised clinically (37% vs 5%) [15] because some have been misclassified as retinal whitening.

### Vessel abnormalities

In CM retinal vessels can have abnormalities within the blood column. It should be remembered that normally retinal vessel walls are transparent and it is the blood column that is seen on funduscopy. The vessels can be orange; FA and histology studies of specifically affected vessels have demonstrated that orange vessels have sequestered pRBCs narrowing the lumen, but that normal RBCs are still able to move within the lumen [16]. White vessels are occluded and are not perfused on FA [14]. White capillaries and post-capillary venules can be seen particularly with a magnifying indirect lens. Histopathology reveals that white vessels are packed with ruptured RBC fragments, haemozoin (parasite-digested haemoglobin) and fibrin polymers, with intact uninfected RBCs absent [16].

Vessels can also have tram lining within the blood column or pale irregularities along the vessel margin. On FA these are demonstrated as intravascular filling defects and histology demonstrates the sequestration of pRBC’s [16]. The retina is the only part of the CNS where sequestration and its tissue effects are visible in vivo in CM.

### Haemorrhages

Retinal Haemorrhages are blot haemorrhages mostly with white-centres akin to Roth spots (Fig.1), which can be extremely numerous, becoming overlapping and confluent. Histologically the white-centre is fibrinogen and fibrin with monocytes containing phagocytosed haemozoin. Intact pRBCs and vessel remnants are uncommon within the haemorrhage [17]. The fibrin clot appears to form after vessel rupture. The number of retinal haemorrhages correlates with the number of cerebral microhaemorrhages in post-mortem samples [18].

The presence of malarial retinopathy identified by an ophthalmologist using indirect ophthalmoscopy has been shown to be diagnostic of CM in fatal parasitaemic cases with a sensitivity of 95% and specificity of 100% (compared to a specificity of 61% for all available data applied to WHO diagnostic criteria) [19]. This has led to the use of malarial retinopathy to diagnose CM by physicians, some of whom utilise indirect ophthalmoscopy [20]. Using the absence of malarial retinopathy to exclude uncertain cases has been widely used in CM research to improve study power [21].

## Ischaemia in the retina and brain

One of the puzzles of paediatric CM was the rapid and apparently full recovery of most survivors. A minority of patients have ischaemic cerebral injury evident as clinically detectable neurological deficits on recovery, but the average age of paediatric CM is 3–5 years of age so neurological examination is limited. It was only when detailed follow-up studies, mapping neurodevelopment, were conducted that was it demonstrated that 30–50% of survivors had some neurological issue after CM. These included global developmental delay, epilepsy and behavioural problems [8, 9].

FAs of children with CM in Malawi have demonstrated scattered areas of capillary non-perfusion which equate to the clinical finding of retinal whitening (Fig. 2) [14, 17]. Sequestration of pRBCs occurs initially in capillaries and post-capillary venules, so it is logical that the ischaemia is in multiple small zones rather than large blocks of tissue as supplied by arterioles. It is likely that the pattern of ischaemia in the retina is mirrored in the brain. Follow up of patients with hyper-reflectivity on OCT scans due to ischaemia shows retinal thinning and atrophy at one year (Fig. 3) [22]. Patchy non-perfusion is more likely to cause global impairments than localised deficits, analogous to multi-infarct dementia rather than stroke. This is an insight into the pathogenesis of CM available from the retina because of the exquisite detail of retinal imaging.

Histological studies of eyes removed at autopsy have demonstrated that orange retinal vessels have sequestered pRBCs adherent to the endothelium and layering out towards the lumen without occluding it entirely to normal RBCs [16]. As malaria trophozoites consume haemoglobin-producing haemazoin, the presence of pRBCs with no haemoglobin reduces the red intensity of the visible blood column. White vessels are full of red cell fragments and haemozoin and are completely occluded. At this point the parasites within RBCs have matured to schizonts which rupture the RBC releasing merozoites into the circulation. Exactly how the dynamic processes of schizont rupture, vessel occlusion, disseminated intravascular coagulation result in haemorrhage formation is unknown—haemorrhages are not spatially associated with white vessels on the retina.

## Brain swelling and fundus findings

A large autopsy study of paediatric CM in Malawi failed to identify severe brain swelling as a factor in death possibly due to an inherent inability to compare to survivors, but also because the brain was decompressed by removing the skull top within a few hours of death [23]. In contrast, MRI scanning identified brain swelling as common in CM, and severe brain swelling as a critical factor in fatal outcome. Brain swelling in the severest grades on admission was present in 84% of fatalities and 27% of survivors [6].

How sequestration leads to severe brain swelling is not known. Potential mechanisms include breakdown in the blood-brain barrier i.e., vasogenic oedema, and tissue ischaemia leading to cytotoxic oedema (hypoxic injury).

Papilloedema, although non-specific, is an important component of malarial retinopathy because it identifies raised intracranial pressure (ICP) associated with severe brain swelling. The presence of papilloedema with malarial retinopathy increases the relative risk of death by 4.5 fold (95% CI 2.7–7.6) [13]. However mild or early papilloedema is difficult to identify with certainty, especially as disc hyperaemia without swelling occurs in CM; and it is not possible to quantify by clinical examination. Papilloedema may take some time to appear after the rapid onset of coma frequently seen in CM. Optic nerve sheath (ONS) ultrasound has been investigated to identify raised ICP in CM. It identified 49% of patients with dilated ONS diameter, and this was associated with a higher mean lumbar puncture opening pressure (220 vs. 160mmCSF, *P* = 0.002) [24]. Only 45% of patients with dilated ONS had papilloedema. A dilated ONS was associated with gross neurological sequelae but not an increased fatality rate (but numbers were modest). ONS ultrasound is binary (dilation present or absent) and operator dependent.

## Blood-retina barrier in cerebral malaria

**Fig. 4:**
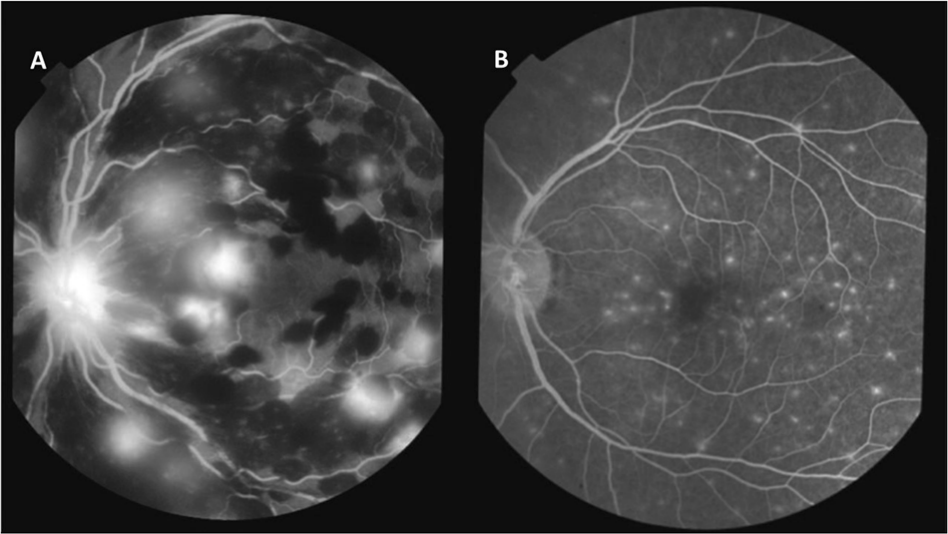
Fluorescein angiograms in cerebral malaria. **A** Multiple large focal leaks and masking from existing retinal haemorrhages. The optic nerve head is also leaking. **B** Multiple punctate leaks in patients with cerebral malaria. Reproduced from MacCormick et al. J Infect Dis. 2022 Mar 15;225(6)1070–1080. 10.1093/infdis/jiaa541.

It is possible to interrogate the blood-retina barrier in CM through FA and by inference draw conclusions about abnormalities in the blood-brain barrier. We have found three patterns of leakage from retinal vessels in CM: vessel leak (Fig. 2), large focal leak and punctate leak (Fig. 4). Punctate leak appears to derive from the retinal pigment epithelium (RPE) or the deep capillary plexus but does not have any clinical corollary. A few (1–5 sites) punctate leaks are present in a quarter of CM patients but only 5% more widely (>5); both severities are associated with death but not neurological sequelae. Large focal leak does have a clinical corollary as FA and colour fundus photographs have clearly demonstrated it to be a retinal haemorrhage in formation. More than 1 large focal leak is again associated with death but not neurological sequelae [17]. Furthermore, we demonstrated with mediation analysis that a large focal leak is consistent with a causal pathway to death via cerebral oedema rather than another direct mechanism.

**Fig. 5:**
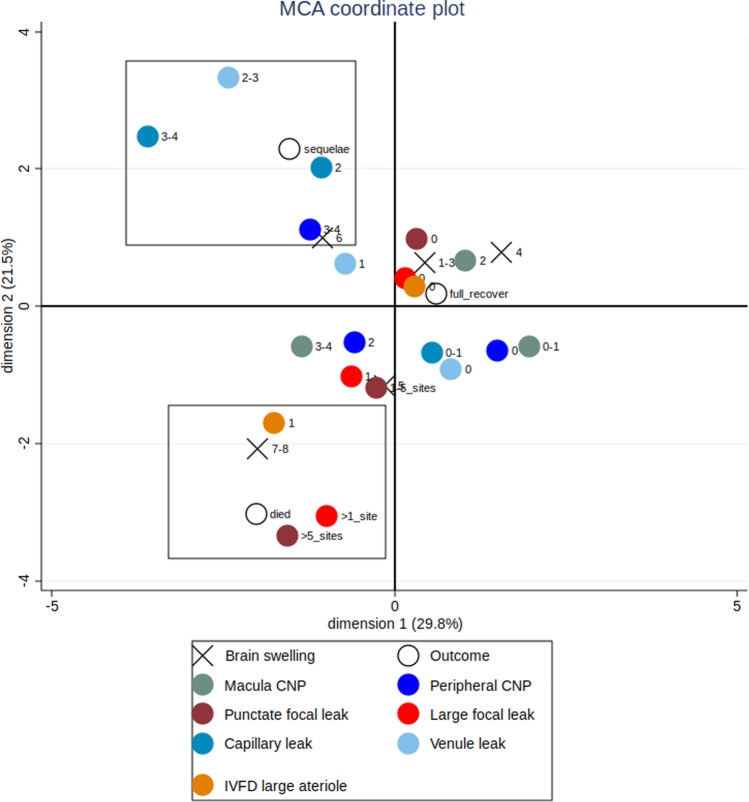
Multiple correspondence analysis (MCA) plot showing fluorescein angiogram features cluster with different outcomes in children with cerebral malaria. This analysis looks for associations in two dimensions, and the boxes are illustrative. Death clusters with large focal leaks (>1 site), punctate leak (>5 sites), arteriolar intravascular filling defects (IVDF), and severe brain swelling (grades 7–8). Neurological sequelae cluster with more severe larger venule leak (grades 2–3) and capillary leak (2 and 3–4), capillary nonperfusion (CNP) in the retinal periphery (3–4) and less severe brain swelling (grade 6). Absent or mild angiographic features, and mild or no brain swelling cluster with full recovery at discharge. Disc leak and large venule IVFD, which were plotted close to the origin, have been omitted from the plot for clarity. Zero indicates the absence of a feature and ascending numbers indicate worsening severity.

In contrast vessel leak, analogous to vasogenic cerebral oedema, was associated in its more severe grades with neurological sequelae, but not death. This is also true for macular capillary non-perfusion. Plotting this on a multiple correspondence analysis, a way of visualising associations in two dimensions, shows two separate clusters (Fig. 5). A cluster including death, severe brain swelling, large focal leak and punctate leak, and a cluster including neurological sequelae, moderate brain swelling, more severe grades of venular and capillary leak and peripheral capillary non-perfusion [17].

Firstly, this implies that neurological sequelae and death are not outcomes on a continuum of increasing severity. They are associated with different blood-retina barrier abnormalities, not increasing the severity of particular abnormalities, suggesting different underlying pathophysiological mechanisms. Sequestration leads to microvascular occlusion, ischaemia and breakdown of vascular integrity. This is associated with moderate brain swelling but this analysis suggests it is survivable with appropriate parasiticidal therapy, albeit with a risk of neurological sequelae from patchy ischaemia. This is a combination of the two traditional mechanisms of brain swelling: vasogenic and cytotoxic oedema.

Secondly, the FA abnormalities associated with death are large focal leak and punctate leak. Large focal leak is a haemorrhage in process captured on a ten-minute FA. Haemorrhage is a physical rupture in the blood-retina barrier, allowing temporary outflow of fluid as well as blood cells. If this is occurring frequently it is indicative of a rapid accumulation of haemorrhages, causing an egress of fluid sufficient to overwhelm compensatory mechanisms and cause severe brain swelling, coning and death.

Punctate leak is not related to retinal sequestration or haemorrhage. It may be an effect of severe systemic metabolic dysfunction (acidosis and/or severe anaemia), or even a pre-agonal event. Systemic metabolic dysfunction may have an effect on the RPE through the choroid which does not suffer significant sequestration.

## Importance and impact

Treatment of CM requires the development of adjunctive therapies beyond the rapid killing of parasites, and indeed a randomised trial is in the process to test two interventions for severe brain swelling: mechanical ventilation and intravenous hypertonic saline (Treating Brain Swelling in Pediatric Cerebral Malaria NCT03300648). However, if researchers want to prevent severe brain swelling rather than mitigate it, our retinal studies suggest that attention should be focused on the mechanism of microhaemorrhages formation including schizont rupture, capillary fragility and disseminated intravascular coagulation [2, 25]. This could identify a target or targets to prevent multiple capillary ruptures and the associated fluid outflow into cerebral tissue.

Secondly FA data suggests that addressing brain swelling may not impact on neurological sequelae which develops through a different mechanism of patchy ischaemia. Separate adjunctive therapies are required to limit ischaemic injury.

## Future work

The single centre Treating Brain Swelling study requires an MRI brain scan to identify children with severe brain swelling as one of its inclusion criteria. Almost no children with CM have access to MRI scanning. If treatment of severe brain swelling is to be trialled elsewhere or implemented more widely in Africa, a more sustainable way of identifying those patients is needed. We are investigating whether OCT of the ONH through quantifying ONH swelling can identify patients with severe brain swelling (OCT in CM study; ISRCTN registry: 11735871). This approach has already shown promise in adult patients with idiopathic intracranial hypertension [26] and children undergoing cranial surgery [27]. Furthermore we, in collaboration with the University of Liverpool Department of Electrical Engineering, are developing a robust, low-cost handheld OCT. We also aim to incorporate automated analysis by artificial intelligence to guide physicians who will be making these treatment decisions.

The OCT in CM study includes two more speculative approaches to brain swelling. One is to measure arteriole: venous width ratio through a high resolution black and white fundus video camera (Epicam®, Epipole, Rosyth, UK). This has been shown to detect change in ICP in patients with continuous intracranial pressure monitoring in Denmark [28], and is potentially more rapidly responsive than papilloedema or ONH swelling which requires inhibited axonal transport. The other is to more accurately enumerate retinal haemorrhages with Epicam fundus imaging. Although higher numbers of haemorrhages have been associated with death [13], that was on a crude ordinal scale. By scaling the number of haemorrhages more accurately on admission we will investigate whether they can identify or predict severe brain swelling as identified by the MRI brain gold standard.

The other major focus of the OCT in CM study is to identify and quantify ischaemia in the retina, demonstrated as hyper-reflectivity, and whether this will predict neurological outcomes after CM. As neurological sequelae can be in the form of developmental delay, and not evident by gross neurological examination, participants will have detailed developmental assessments at one and two years and compared to local controls. If macula OCT can identify children at high risk of neurological sequelae there are supportive interventions which can benefit child and family [10]. In addition it will add to the impetus for research into interventions which will limit ischaemic brain injury and benefit survivors.

## Summary

CM is a common cause of death of children in Africa, and will remain so despite the use of insecticide-treated bednets and implementation of new partially effective vaccines. CM is accompanied by distinctive retinal abnormalities, termed malarial retinopathy which is diagnostic and prognostic. Malarial retinopathy provides a keyhole view of the cerebral pathophysiology of CM allowing in vivo studies which are not possible in any other way.

We have used fluorescein angiography and retinal histology to demonstrate patchy capillary non-perfusion and ischaemia. In the brain, patchy ischaemia may be a cause of global cerebral impairments in survivors. We have also investigated the blood-retina barrier to understand mechanisms of brain swelling. Although ischaemia and vascular leakage are associated with mild to moderate brain swelling, severe brain swelling and death are associated with the rapid accumulation of retinal white-centred blot haemorrhages. A haemorrhage is a physical breach in the blood-tissue barrier with accompanying uncontrolled outflow of cells and fluid into the tissue. If a multitude of haemorrhages are occurring in a short time, our research suggests this pushes patients to severe brain swelling, coning and death.

Current research is focused on using a low-cost handheld OCT to identify patients with severe brain swelling and survivors at risk of brain injury. Future research is needed to identify mechanisms of haemorrhage and targets for earlier intervention with adjunctive therapies.
